# The *Tabby* cat locus maps to feline chromosome B1

**DOI:** 10.1111/j.1365-2052.2006.01458.x

**Published:** 2006-08

**Authors:** L A Lyons, S J Bailey, K C Baysac, G Byrns, C A Erdman, N Fretwell, L Froenicke, K W Gazlay, L A Geary, J C Grahn, R A Grahn, G M Karere, M J Lipinski, H Rah, M T Ruhe, L H Bach

**Affiliations:** * Population Health and Reproduction, School of Veterinary Medicine, University of California – Davis Davis, CA 95616, USA; † WALTHAM Centre for Pet Nutrition, Melton Mowbray Leicestershire, UK; ‡ Veterinary Genetics Laboratory, School of Veterinary Medicine, University of California – Davis Davis, CA 95616, USA

**Keywords:** Abyssinian, blotched, cat, classic, *Felis catus*, mackerel, *Tabby*

## Abstract

The *Tabby* markings of the domestic cat are unique coat patterns for which no causative candidate gene has been inferred from other mammals. In this study, a genome scan was performed on a large pedigree of cats that segregated for *Tabby* coat markings, specifically for the *Abyssinian* (*T*^*a*^-) and *blotched* (*t*^*b*^*t*^*b*^) phenotypes. There was linkage between the *Tabby* locus and eight markers on cat chromosome B1. The most significant linkage was between marker *FCA700* and *Tabby* (*Z* = 7.56, *θ* = 0.03). Two additional markers in the region supported linkage, although not with significant LOD scores. Pairwise analysis of the markers supported the published genetic map of the cat, although additional meioses are required to refine the region. The linked markers cover a 17-cM region and flank an evolutionary breakpoint, suggesting that the *Tabby* gene has a homologue on either human chromosome 4 or 8. Alternatively, *Tabby* could be a unique locus in cats.

Coat colour striping patterns, commonly known as *Tabby* markings, are frequent in domestic cats, but a candidate is not available from other species. Traditionally, three autosomal alleles have been suggested for *Tabby*: *Abyssinian* (*T*^*a*^; a.k.a. *ticked*), *mackerel* (*T*^*m*^; a.k.a. *striped*) and *blotched* (*t*^*b*^; a.k.a. *classic*). Cats with traditional patterns resulting from *Tabby* alleles are presented in Fig. 1. The allelic series *T*^*a*^ > *T*^*m*^ > *t*^*b*^ has been suggested; however, this series is disputed because the *Tabby* coat patterns that form a distinctive spotted pattern cannot be explained with a single, allelic model. However, traditional patterns are represented by three alleles: *T*^*a*^, which produces few markings and possible stripes on the head, legs and tail but not on the torso; *T*^*m*^, which produces stripes on the head, legs, tail and torso; and *t*^*b*^, which produces stripes on the head, legs and tail but circular patterns on the torso. Cats that are homozygous for the *Abyssinian* allele (*T*^*a*^*T*^*a*^) may have less barring on the legs and can be distinguished from heterozygotes. Hence, the *T*^*a*^ allele can be considered co-dominant to *T*^*m*^ and *t*^*b*^ when considering the leg barring, but this demarcation has variable expression and is difficult to distinguish.

**Figure 1:**
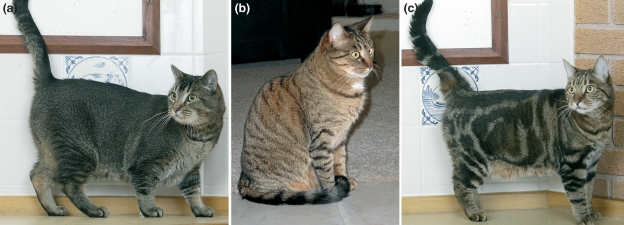
*Tabby* patterns in the domestic shorthair cats: (a) *Abysinnian*, *T*^*a*^-, (b) *mackerel*, *T*^*m*^*T*^*m*^ or *Tt*^*b*^, and (c) *blotched*, *t*^*b*^*t*^*b*^. The *Abysinnian* pattern is also known as *ticked*, the *mackerel* pattern as *striped* and the *blotched* pattern as *classic*. Homozygous *T*^*a*^*T*^*a*^ individuals have the same pattern on the torso but may have less barring on the legs and tail. The *T*^*m*^*t*^*b*^*mackerel* pattern may appear as the *broken mackerel* or *spotted mackerel* patterns; however, these phenotypes have not segregated in pedigrees, and the *spotted* pattern may be affected by modifiers.

In the mouse, loss-of-function alleles for the sex-linked ectodysplasin-A (*Eda*) gene were originally described as ‘Tabby’, but this phenotype affects hair structure and is completely unrelated to the *Tabby* phenotypic patterns in cats (Ferguson *et al.* 1997; Srivastava *et al.* 1997). Therefore, a full-genome scan across the cat autosomes was performed to identify the locus for *Tabby* markings. An extended pedigree of 64 cats segregating for *Tabby* markings (Fig. 2) from the WALTHAM Centre for Pet Nutrition (Melton Mowbray, Leics, UK) was used for linkage analysis. Three marking patterns were used to demarcate the *Tabby* phenotypes: *Abyssinian*, *mackerel* and *blotched* (Fig. 1; Lomax & Robinson 1988). Buccal cells were non-invasively obtained by swabbing the internal cheek of each cat with a cytological brush. DNA was extracted from buccal cells from the cats representing the pedigree as previously described (Oberbauer *et al.* 2003).

**Figure 2:**
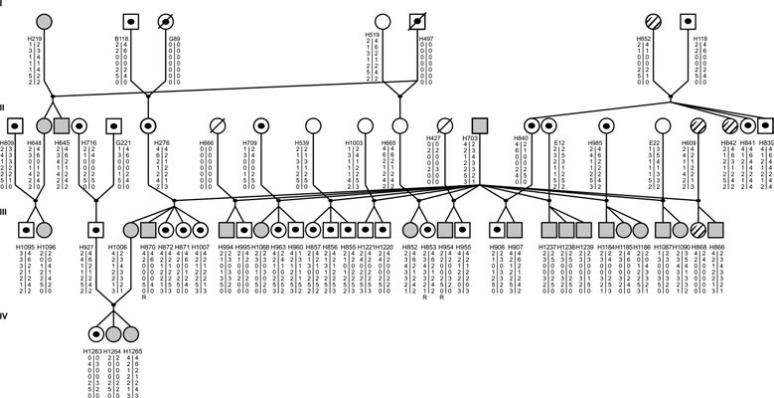
Pedigree segregating for domestic cat *Tabby* patterns and linked markers from cat chromosome B1. Circles represent females, squares represent males, filled symbols indicate phenotypically *Tabby Abyssinian* cats, symbols with a center dot are *Tabby blotched* patterned cats and striped symbols indicate *Tabby mackerel* pattern cats. Unknown patterns are presented as open symbols. Unknown cats had solid, *non-agouti* colours, thus *Tabby* patterns could not be distinguished. Symbols with a diagonal slash represent cats that were not available for the analyses. Numbers under the symbols represent the laboratory sample numbers. Genotypes for the seven linked markers (*FCA023*, *FCA809*, *FCA811*, *FCA812*, *FCA700*, *FCA254* and *FCA813*) are represented below the cat identification numbers respectively and are presented as haplotypes. No genotype data are represented by ‘0’. Cat H118 was duplicated to break an inbreeding loop and is also represented as B118. Cats that are recombinants between at least one marker in the haplotype and the *Tabby* phenotype are indicated with an ‘R’.

Approximately 150 feline-derived microsatellites were selected from the feline linkage (Menotti-Raymond *et al.* 1999, 2003) and radiation hybrid (RH) (Murphy *et al.* 2000) maps to conduct a genome scan with an average marker spacing of 75 cR. Genotyping for the markers (Young *et al.* 2005) and two-point linkage between the microsatellite genotypes and the *Tabby* phenotype (Lyons *et al.* 2005) was conducted using the linkage software program (Lathrop *et al.* 1984). Only torso patterns were considered for the phenotypic determination of *Tabby Abyssinian* to avoid problems when determining homozygotes and heterozygotes for this allele. To represent the dominant allelic series, the *Tabby* alleles were coded as binary factors for the linkage analysis: 111 representing *T*^*a*^, 011 representing *T*^*m*^ and 001 representing the *t*^*b*^ allele. Frequencies of the phenotype and of the co-dominant marker alleles were estimated by directly counting unrelated parents in the pedigree. One individual (H118) was duplicated to remove an inbreeding loop.

Pairwise analyses for markers on cat chromosome B1 and the *Tabby* phenotype are presented in Table 1. Linkage was suggested between eight markers on feline chromosome B1 and *Tabby* with LOD scores >3.0. Seven additional markers on cat chromosome B1 were genotyped to refine the linked region and the recombination map for this chromosome (Table 1). The most significant linkage was between marker *FCA700* and *Tabby* (*Z* = 7.56, *θ* = 0.03). The small number of meioses in the pedigree restricts accurate multipoint mapping of the region; however, the *Tabby* locus likely resides between markers *FCA559* and *FCA254*, which cover an estimated 113.6-cR region on the RH map of cat chromosome B1 and approximately 15.9 Mb or 17 cM on the linkage map (Menotti-Raymond *et al.* 2003). The other 55 markers genotyped across the pedigree were not linked to *Tabby* (data not shown).

**Table 1:** LOD scores for pairwise comparisons between *Tabby* and chromosome B1 microsatellite markers.

Marker	Feline B1 position (cR)^1^	HSA chromosome^2^	Max LOD (*Z*)^3^	Recombination rate (*θ*) at LOD (*Z*)
*FCA577*	156.7	No RH	NS	–
*FCA519*	248.7	8	1.42	0.191
*FCA559*	416.9	8	3.17	0.099
*FCA339*	566.1	8	–	–
*FCA023*	597.1	No RH	5.38	0.038
*FCA809*	608.7	8	5.34	0.081
*FCA810*	618.1	No RH	0.48	0.000
*FCA811*	630.2	No RH	3.69	0.048
*FCA812*	648.1	No RH	3.76	0.053
*FCA700*	663.0	4	7.56	0.030
*FCA254*	679.7	No RH	5.09	0.106
*FCA813*	701.2	No RH	3.68	0.05
*FCA550*	731.8	No RH	NS	–
*FCA814*	774.0	No RH	NS	–
*FCA815*	783.6	No RH	–	–
*FCA513*	793.6	No RH	2.64	0.144
*FCA816*	826.1	4	NS	–

1 Radiation hybrid (RH) positions were previously published (Menotti-Raymond et al. 2003).

2 Human chromosome that has homologous sequence to the feline sequence. Markers without a chromosome position have not been assigned on the human radiation hybrid map (no RH).

3 NS, non-significant LOD score.

Positions (in cR) of the markers on cat chromosome B1 and the homologous sequences in humans are shown in Table 1. The comparative map between the cat and human indicates that a gene controlling the *Tabby* locus is on feline chromosome B1 near a breakpoint of evolutionarily conserved segments that are homologous to human chromosomes 4 and 8.

Domestic cats have several tabby coat patterns, including *Abyssinian*, *mackerel*, *broken mackerel*, *spotted* and *blotched*. The analysed pedigree was limited primarily to the *Abyssinian* and *blotched* phenotypes. One mating between phenotypically *Abyssinian* and *mackerel* cats produced one cat of each phenotype and one mating between phenotypically *blotched* and *mackerel* cats produced two *blotched* and one *mackerel* cat. The segregation of these patterns is consistent with the hypothesis of a tri-allelic locus with a dominant series of alleles; however, the number of meioses from *mackerel* patterned cats was limited in our pedigree. Leg barring was not used to distinguish *Abyssinian* heterozygotes, and the *mackerel* patterns were not delineated between *mackerel* and *broken mackerel* in the analysis. Thus, controlled matings are required to clarify these classifications. In wild felids, such as lions and pumas, spotted *Tabby* patterns are evident in cubs but are lacking in the *TabbyAbyssinian* patterns of the adults. Along with the presentation of other coat pattern markings such as the *spotted patterns* and *broken mackerel* patterns, alternative hypotheses for the dominance of alleles and the presence of potential modifiers are still possible. The identified 17-cM region on cat chromosome B1 should be explored for candidate genes and causative mutations for feline *Tabby* patterns.
